# The calcium-sensing receptor in sepsis and septic shock, mechanistic pathways and translational perspectives: a systematic review

**DOI:** 10.1007/s00011-026-02227-4

**Published:** 2026-04-17

**Authors:** Valentine Janssen, Saïd Kamel, Michel Slama, Osama Abou-Arab, Michel Brazier, Romuald Mentaverri, Aurélien Mary

**Affiliations:** 1 MP3CV Laboratory-UPJV-UR 7517, University of Picardie Jules Verne, Amiens, France; 2 Amiens – Picardie University Hospital, Amiens, France

**Keywords:** Calcium-sensing receptor, Cytokines, Inflammation, Sepsis, Septic shock

## Abstract

**Background:**

Sepsis and septic shock are major causes of mortality in critically ill patients. They are linked to widespread metabolic and immune dysregulation, including alterations in calcium and phosphate homeostasis. The calcium-sensing receptor (CaSR) not only plays a role in mineral balance, but also modulates key immune pathways and may contribute to the pathogenesis of sepsis.

**Methods:**

A systematic literature review was conducted according to PRISMA 2020 guidelines. Four databases (PubMed, EMBASE, Cochrane Library, and Google Scholar) were searched for studies published since 1990. To be eligible for inclusion, articles had to be original research, reviews, or clinical trials involving adult models (mammalian or human). Full-text availability was required. Risk of bias was assessed for all studies included.

**Results:**

Sixty-six articles met the inclusion criteria: 49 original studies and 17 reviews. No randomized controlled trials or meta-analyses were identified. Most studies relied on in vitro or in vivo models. CaSR was consistently reported to be upregulated or activated following exposure to bacterial and inflammatory stimuli in immune cells, including monocytes and lymphocytes. CaSR activation promotes proinflammatory cytokine release, notably IL-1β via the NOD like receptor family 3 (NLRP3) inflammasome although results in intestinal epithelial models remain inconsistent. In non-septic models, CaSR activation was associated with tissue and organ injury, including renal and cardiac damage, as well as vasoplegia related to endothelial dysfunction. Preclinical models of pneumonia and endotoxemia suggest that CaSR antagonists may effectively mitigate inflammation and organ injury.

**Conclusion:**

This systematic review identifies the CaSR as an amplifier of the host inflammatory response across both septic and non-septic preclinical models The lack of robust clinical data underscores the need for translational studies assessing CaSR expression or activity in patients with sepsis or septic shock, alongside in vivo validation of CaSR inhibition as a therapeutic strategy.

**Supplementary Information:**

The online version contains supplementary material available at 10.1007/s00011-026-02227-4.

## Introduction

Septic shock is a life-threatening clinical syndrome contributing to the global burden of sepsis. It accounts for approximately 11 million deaths worldwide annually, representing one in five of all deaths [1]. According to the SEPSIS-3 international consensus, septic shock is defined as a dysregulated host response to infection resulting in life-threatening organ dysfunction [2]. Its pathophysiology involves two initial and interdependent phases: pathogen invasion and an excessive systemic immune response. Current standard-of-care treatments include early administration of appropriate anti-infective agents, fluid resuscitation and vasopressor support to maintain tissue perfusion, and advanced organ support [2]. Although modulation of inflammation appears to be an attractive therapeutic target, the efficacy of hydrocortisone remains contested [3, 4] as does the role of personalized immunotherapies [5]. As a result, no immunomodulatory therapy has yet been formally approved for the treatment of septic shock.

Severe sepsis is associated with profound alterations in calcium-phosphate homeostasis, including hypocalcemia [6, 7] secondary hypoparathyroidism, and vitamin D deficiency [8]). These alterations suggest a potential mechanistic link between the inflammatory response in sepsis and dysregulated phosphate and calcium homeostasis. Macromolecules involved in regulating levels of these micronutrients could therefore be interesting therapeutic targets.

The calcium-sensing receptor (CaSR), a class C G-protein-coupled receptor discovered in 1993, is primarily recognized for its regulatory functions in the parathyroid glands and kidneys. In response to elevated extracellular calcium concentrations, it suppresses parathyroid hormone (PTH) secretion and inhibits renal calcium reabsorption, thereby promoting hypocalcemia [9]. CaSR is also expressed in immune cells and hematopoietic progenitors [10], where it can have an influence beyond its endocrine role. The first link between CaSR and NLRP3 inflammasome regulation was established by Lee et al. [11]. Building on this, Canton et al. [12] showed that CaSR enables constitutive macropinocytosis in human monocytes and is essential for the cytosolic delivery of microbial ligands to pattern-recognition receptors. Together, these seminal studies established CaSR as a key immunomodulatory node, prompting further investigation into its role in systemic inflammatory disorders, such as rheumatoid arthritis [13], atherosclerosis and type 2 diabetes [14].

CaSR can be pharmacologically modulated using calcimimetics – positive allosteric modulators approved for the treatment of hyperparathyroidism – and calcilytics – negative allosteric modulators that are being explored in the context of osteoporosis and chronic inflammatory conditions. However, no CaSR-targeting agent is currently approved for the treatment of sepsis or septic shock. Based on its dual role in calcium homeostasis and immune system regulation, we hypothesized that the CaSR may be a key pathophysiological mediator in septic shock. We conducted this systematic review to assess the current state of knowledge on the involvement of CaSR in sepsis and septic shock, and to explore its potential as a therapeutic target.

## Materials and methods

### 2020 PRISMA reviewing method

The reviewing method applied followed the conventional PRISMA method [15]. The review was registered in PROSPERO (registration number: CRD42024536199) before starting data extraction. The review was initiated in April 2024, and the last search in databases was carried out in December 2024 (at which time data were frozen). Our research strategy involved screening articles contained in Google Scholar, PubMed, Elsevier EMBASE and the Cochrane Library.

The databases were queried using three groups of keywords:

- Set 1- (Receptors, Calcium-Sensing [MeSH Terms] OR “Calcium sensing receptor” OR “CaSR” OR “Ca2+-sensing receptor” OR “calcium receptor”) AND (Shock, Septic[MeSH Terms], “Septic shock” OR “Endotoxic shock” OR “Sepsis-induced hypotension” OR “Refractory septicemia” OR “Vasopressor-resistant septicemia” OR “Severe Sepsis” OR “septicemic shock” OR “sepsis” OR “bloodstream infection”);
- Set 2- (Receptors, Calcium-Sensing [MeSH Terms] OR “Calcium sensing receptor” OR “CaSR” OR “Ca2+-sensing receptor” OR “calcium receptor”) AND (Inflammation[MeSH Terms]);
- Set 3- (Receptors, Calcium-Sensing [MeSH Terms] OR “Calcium sensing receptor” OR “CaSR” OR “Ca2+-sensing receptor” OR “calcium receptor”) AND cytokine[MeSH Terms]).

The first set of keywords was considered to facilitate the identification of articles directly related to the core topic, whereas the other two aimed to broaden the scope of the searches to encompass inflammatory processes. In all cases, the subsequent analysis of references identified was conducted within the context of sepsis, by identifying studies that directly combined a sepsis model with CaSR modulation. Relevant articles were selected based on the inclusion and exclusion criteria.

### Inclusion and exclusion criteria

The inclusion criteria applied were as follows: publication type (randomized clinical trials (RCT)), clinical trials, original research articles (in vivo, in vitro), systematic reviews, reviews, meta-analyses), adult population or sepsis in mammalian species, full-text availability (either through university access or by contacting the corresponding authors), and publication date after 1990. Exclusion criteria were: absence of any direct link to or interpretation related to septic shock, publication type (case reports, case series, guidelines, recommendations), population type (children, young animals, non-mammalian species, clinical trials with non-septic patients).

### Bias assessment and article categorization

Bias was monitored using methods adapted to the study type. Thus, RCT and clinical trials were evaluated using the Jadad scale [16], whereas systematic reviews were assessed by applying the AMSTAR method [17]. For in vitro studies, parameters such as study methodology (cell types, animal models, use of CaSR modulators), quality of experiments, and potential bias were examined. Each main finding – the signaling pathway involved, pro- or anti-inflammatory effects, tissue of origin, modulation of CaSR expression, cytokine production – was categorized to analyze consistency and homogeneity. Articles were also classed in one of two categories: those directly involving a septic shock model, and those addressing indirect mechanistic aspects. Throughout the review process, two reviewers independently selected and categorized articles. Any disagreements were consensually resolved through adjudication by a third reviewer.

### Statistical considerations for calcilytic dosing

Calcilytic doses reported in the included studies were compiled and analysed. The descriptive median dose and its 95% confidence interval were estimated using a nonparametric bootstrapping method based on 10,000 times repeated random resampling with replacement to generate an empirical distribution of the median.

### Assessment of homogeneity across extracted data

Given the nature of the data extracted – categorical classifications of study conclusions –classical homogeneity tests comparing independent groups could not be applied. Instead of performing formal statistical analyses, we conducted a descriptive thematic synthesis of the articles’ findings to avoid over interpretation.

### Large language model use

A large language model (OpenAI, GPT-5.2, accessed January 2026) was used to assist in rephrase the manuscript. All output was critically reviewed, modified, and validated by the authors to ensure scientific accuracy and coherence and absence of hallucination. The large language model was not involved in the retrieval or analysis of papers, nor in the thematic classification.

## Results

**Fig. 1:**
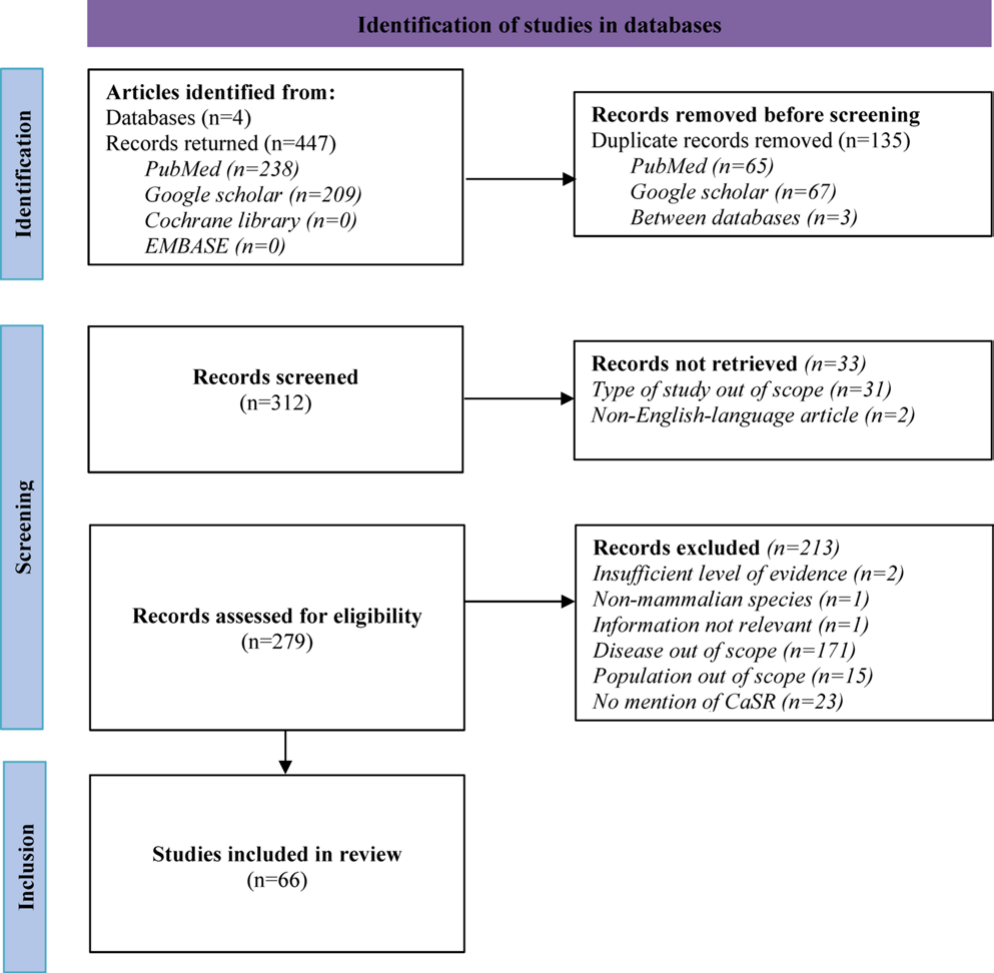
Flow chart of selection of studies for inclusion in the systematic review, based on the PRISMA method. In total, 66 articles were included in the review. CaSR = Calcium-sensing receptor

**Fig. 2:**
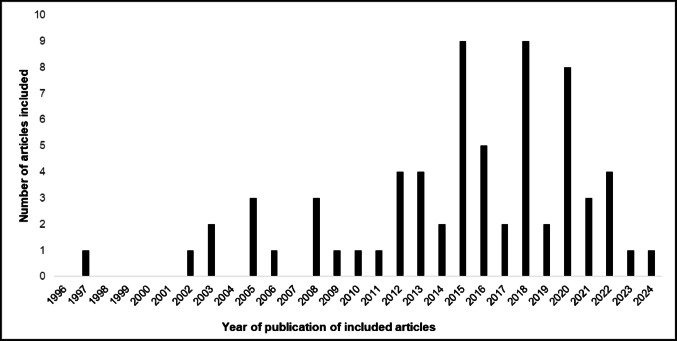
Distribution of the articles included in the systematic review by publication year. The majority (*n* = 54 (82%)) of the articles included were published between 2010 and 2024

A total of 447 records were identified through database searches (PubMed: 238, Google Scholar: 209, Cochrane Library: 0, EMBASE: 0) (Fig. 1). After removing 135 duplicates, the remaining 312 records were screened. A further 33 articles were not considered relevant, either because their type of article were out of scope (not related to sepsis, *n* = 31) or because they were not published in English (*n* = 2). The resulting 279 records were then assessed. Eligibility assessment led to the exclusion of 213 articles, leaving 66 studies for inclusion in the final review, all of which were available as full-texts. The 66 articles included comprised 49 original research articles (Table 1) and 17 narrative (i.e., non-systematic) reviews (Table 2). The median year of publication for the studies included was between 2015 and 2016, with publication peaks observed in 2015 and 2018 (Fig. 2).

**Table 1:** References of original research articles included in the review

Keywords used for search strategy	Database	DOI	Reference (Title, Authors, Journal)	Year of publication	Population type	Direct CaSR-sepsis scope	In vitro	In vivo	Clinical
3. Cytokines	PubMed	10.1038/ncomms2339	“Extracellular Ca2 + is a danger signal activating the NLRP3 inflammasome through G protein-coupled calcium sensing receptors”, Rossol M, Pierer M, Raulien N, Quandt D, Meusch U, Rothe K, Schubert K, Schöneberg T, Schaefer M, Krügel U, Smajilovic S, Bräuner-Osborne H, Baerwald C, Wagner U., Nat Commun. [18]	2012	Mice (GPRC6 -/- mice), human donor	X	X	X	
3. Cytokines	PubMed	10.1038/nature11588	“The calcium-sensing receptor regulates the NLRP3 inflammasome through Ca2 + and cAMP”, Lee GS, Subramanian N, Kim AI, Aksentijevich I, Goldbach-Mansky R, Sacks DB, Germain RN, Kastner DL, Chae JJ., Nature. [11]	2012	Human cells (PBMC)	X	X		X
3. Cytokines	PubMed	10.1016/j.molimm.2012.09.010	“Expression of the calcium sensing receptor in human peripheral blood T lymphocyte and its contribution to cytokine secretion through MAPKs or NF-κB pathways”, Li T, Sun M, Yin X, Wu C, Wu Q, Feng S, Li H, Luan Y, Wen J, Yan L, Zhao B, Xu C, Sun Y., Mol Immunol. [19]	2013	Human cells (lymphocytes from PBMC)	X	X		X
3. Cytokines	PubMed	10.1007/s11033-014-3296-1	“Different mechanism of LPS-induced calcium increase in human lung epithelial cell and microvascular endothelial cell: a cell culture study in a model for ARDS”, Zhang K, Wang P, Huang S, Wang X, Li T, Jin Y, Hehir M, Xu C., Mol Biol Rep. [20]	2014	Human cells (ARDS model)	X	X		
1. Sepsis and septic shock	PubMed	10.1016/j.molimm.2014.10.018	“Activation of calcium-sensing receptor increases TRPC3/6 expression in T lymphocyte in sepsis”, Wu QY, Sun MR, Wu CL, Li Y, Du JJ, Zeng JY, Bi HL, Sun YH., Mol Immunol. [21]	2015	In vitro study (mammalian cells) (in vivo mouse LPS model - lymphocyte)	X	X	X	
1. Sepsis and septic shock	PubMed	10.1016/j.molimm.2014.08.007	“Calcium-sensing receptor in the T lymphocyte enhanced the apoptosis and cytokine secretion in sepsis”, Wu CL, Wu QY, Du JJ, Zeng JY, Li TT, Xu CQ, Sun YH., Mol Immunol.[22]	2015	In vitro study (mammalian cells) (in vivo mouse LPS model - lymphocyte)	X	X	X	
1. Sepsis and septic shock	PubMed	10.1021/acs.jafc.7b02109	“Intervention of Dietary Dipeptide Gamma-l-Glutamyl-l-Valine (γ-EV) Ameliorates Inflammatory Response in a Mouse Model of LPS-Induced Sepsis”, Chee ME, Majumder K, Mine Y., J Agric Food Chem. [23]	2017	Mouse model of sepsis	X		X	
3. Cytokines	PubMed	10.1016/j.molimm.2017.07.012	“NPS 2143, a selective calcium-sensing receptor antagonist inhibits lipopolysaccharide-induced pulmonary inflammation”, Lee JW, Park HA, Kwon OK, Park JW, Lee G, Lee HJ, Lee SJ, Oh SR, Ahn KS., Mol Immunol. [24]	2017	Intranasal LPS - induced inflammatory mice	X	X	X	
2. Inflammation	PubMed	10.4049/jimmunol..1701212	,“Staphylococcal Superantigens Use LAMA2 as a Coreceptor To Activate T Cells”, Li Z, Zeppa JJ, Hancock MA, McCormick JK, Doherty TM, Hendy GN, Madrenas J., J Immunol. [25]	2018	Mice and in vitro analysis	X	X	X	
2. Inflammation	PubMed	10.1091/mbc.E17-06-0419	“Differential ability of proinflammatory and anti-inflammatory macrophages to perform macropinocytosis”, Redka DS, Gütschow M, Grinstein S, Canton J., Mol Biol Cell. [26]	2018	Human cells (PBMC)	X	X		
3. Cytokines	PubMed	10.3389/fimmu.2020.570872	“Prokineticin 2 via Calcium-Sensing Receptor Activated NLRP3 Inflammasome Pathway in the Testicular Macrophages of Uropathogenic Escherichia coli-Induced Orchitis”, Su Y, Zhang Y, Hu Z, He L, Wang W, Xu J, Fan Z, Liu C, Zhang H, Zhao K., Front Immunol. [27]	2020	E.coli-induced orchitis, rats	X		X	
2. Inflammation	PubMed	10.3389/fimmu.2021.748497	“Tryptophan Ameliorates Barrier Integrity and Alleviates the Inflammatory Response to Enterotoxigenic Escherichia coli K88 Through the CaSR/Rac1/PLC-γ1 Signaling Pathway in Porcine Intestinal Epithelial Cells”, Liu G, Gu K, Wang F, Jia G, Zhao H, Chen X, Wu C, Zhang R, Tian G, Cai J, Tang J, Wang J., Front Immunol. [28]	2021	Mammalian cells	X	X		
3. Cytokines	PubMed	10.1006/bbrc.1997.7207	“Inhibition of PTH secretion by interleukin-1 beta in bovine parathyroid glands in vitro is associated with an up-regulation of the calcium-sensing receptor mRNA”, Nielsen PK, Rasmussen AK, Butters R, Feldt-Rasmussen U, Bendtzen K, Diaz R, Brown EM, Olgaard K., Biochem Biophys Res Commun. [29]	1997	Bovine cells (PT cells)		X		
3. Cytokines	PubMed	10.1152/ajprenal.00108.2002	“Calcium-sensing receptor-mediated TNF production in medullary thick ascending limb cells”, Wang D, Pedraza PL, Abdullah HI, McGiff JC, Ferreri NR., Am J Physiol Renal Physiol. [30]	2002	Human cells (mTAL)		X		
3. Cytokines	PubMed	10.1677/jme.0.0310609	“Parathyroid hormone (PTH) secretion, PTH mRNA and calcium-sensing receptor mRNA expression in equine parathyroid cells, and effects of interleukin (IL)-1, IL-6, and tumor necrosis factor-alpha on equine parathyroid cell function”, Toribio RE, Kohn CW, Capen CC, Rosol TJ., J Mol Endocrinol. [31]	2003	Equine cells (PT cells)		X		
3. Cytokines	PubMed	10.1002/jcb.20511	“Roles of Ca2 + and the Ca2+-sensing receptor (CASR) in the expression of inducible NOS (nitric oxide synthase)-2 and its BH4 (tetrahydrobiopterin)-dependent activation in cytokine-stimulated adult human astrocytes”, Dal Pra I, Chiarini A, Nemeth EF, Armato U, Whitfield JF., J Cell Biochem. [32]	2005	Human cells (astrocytes)		X		
3. Cytokines	PubMed	10.1074/jbc.M408587200	“Calcium-sensing receptor gene transcription is up-regulated by the proinflammatory cytokine, interleukin-1beta. Role of the NF-kappaB pathway and kappaB elements”, Canaff L, Hendy GN., J Biol Chem. [33]	2005	Human cells (HCKC cells and TT cells)		X		
3. Cytokines	Google Scholar	10.1161/01.RES.0000178787.59594.a0	“Evidence in favor of a calcium-sensing receptor in arterial endothelial cells: studies with calindol and Calhex 231”, Weston AH, Absi M, Ward DT, Ohanian J, Dodd RH, Dauban P, Petrel C, Ruat M, Edwards G., Circulation research. [34]	2005	Mice		X	X	
3. Cytokines	PubMed	10.1152/ajprenal.00223.2005	“NFAT regulates calcium-sensing receptor-mediated TNF production”, Abdullah HI, Pedraza PL, Hao S, Rodland KD, McGiff JC, Ferreri NR., Am J Physiol Renal Physiol. [35]	2006	Human cells (mTAL)		X		
3. Cytokines	PubMed	10.1152/ajprenal.90316.2008	“Calcium-sensing receptor signaling pathways in medullary thick ascending limb cells mediate COX-2-derived PGE2 production: functional significance”, Abdullah HI, Pedraza PL, McGiff JC, Ferreri NR., Am J Physiol Renal Physiol. [36]	2008	Rats		X	X	
3. Cytokines	PubMed	10.1074/jbc.M708087200	“The proinflammatory cytokine, interleukin-6, up-regulates calcium-sensing receptor gene transcription via Stat1/3 and Sp1/3”, Canaff L, Zhou X, Hendy GN., J Biol Chem. [37]	2008	Human cells (HCK cells)		X		
3. Cytokines	PubMed	10.1152/ajprenal.00509.2006	“CaR activation increases TNF production by mTAL cells via a Gi-dependent mechanism”, Abdullah HI, Pedraza PL, McGiff JC, Ferreri NR., Am J Physiol Renal Physiol. [38]	2008	Human cells (mTAL)		X		
3. Cytokines	PubMed	10.1152/ajprenal.90436.2008	“Expression and function of NFAT5 in medullary thick ascending limb (mTAL) cells”, Hao S, Zhao H, Darzynkiewicz Z, Battula S, Ferreri NR., Am J Physiol Renal Physiol. [39]	2009	Human cells (mTAL)		X		
3. Cytokines	PubMed	10.1007/s11010-010-0489-3	“The functional expression of calcium-sensing receptor in the differentiated THP-1 cells”, Xi YH, Li HZ, Zhang WH, Wang LN, Zhang L, Lin Y, Bai SZ, Li HX, Wu LY, Wang R, Xu CQ., Mol Cell Biochem. [40]	2010	Human cells (THP-1)		X		
3. Cytokines	Google Scholar	10.1161/CIRCRESAHA.112.266361	“Enhanced Ca2+-sensing receptor function in idiopathic pulmonary arterial hypertension”, Yamamura A, Guo Q, Yamamura H, Zimnicka AM, Pohl NM, Smith KA, Fernandez RA, Zeifman A, Makino A, Dong H., Circulation research. [41]	2012	Human cells (arterial smooth muscle cells)		X		
3. Cytokines	PubMed		“Altered monocyte calcium-sensing receptor expression in patients with type 2 diabetes mellitus and atherosclerosis”, Malecki R, Fiodorenko-Dumas Z, Jakobsche-Policht U, Malodobra M, Adamiec R., J Physiol Pharmacol. [14]	2013	Adult with diabetes and atherosclerosis				X
3. Cytokines	PubMed	10.1371/journal.pone.0074800	“Determination and modulation of total and surface calcium-sensing receptor expression in monocytes in vivo and in vitro”, Paccou J, Boudot C, Mary A, Kamel S, Drüeke TB, Fardellone P, Massy Z, Brazier M, Mentaverri R., PLoS One. [42]	2013	Human cells (PBMC)		X		X
2. Inflammation	PubMed	10.1016/j.febslet.2014.05.007	“Epithelial CaSR deficiency alters intestinal integrity and promotes proinflammatory immune responses”, Cheng SX, Lightfoot YL, Yang T, Zadeh M, Tang L, Sahay B, Wang GP, Owen JL, Mohamadzadeh M., FEBS Lett. [43]	2014	Mice with colitis			X	
2. Inflammation	PubMed	10.1126/scitranslmed.aaa0282	“Calcium-sensing receptor antagonists abrogate airway hyperresponsiveness and inflammation in allergic asthma”, Yarova PL, Stewart AL, Sathish V, Britt RD Jr, Thompson MA, P Lowe AP, Freeman M, Aravamudan B, Kita H, Brennan SC, Schepelmann M, Davies T, Yung S, Cholisoh Z, Kidd EJ, Ford WR, Broadley KJ, Rietdorf K, Chang W, Bin Khayat ME, Ward DT, Corrigan CJ, T Ward JP, Kemp PJ, Pabelick CM, Prakash YS, Riccardi D., Sci Transl Med. [44]	2015	Adults with allergic asthma		X	X	
3. Cytokines	PubMed	10.1038/srep16719	“GPRC6A mediates Alum-induced Nlrp3 inflammasome activation but limits Th2 type antibody responses”, Quandt D, Rothe K, Baerwald C, Rossol M., Sci Rep. [45]	2015	CaSR -/- mice			X	
3. Cytokines	PubMed	10.1021/acs.jafc.5b03812	“Anti-inflammatory Effects of Poly-L-lysine in Intestinal Mucosal System Mediated by Calcium-Sensing Receptor Activation”, Mine Y, Zhang H., J Agric Food Chem. [46]	2015	Human cells (intestinal mucosal system)		X	X	
3. Cytokines	PubMed	10.1159/000374048	“Activation in M1 but not M2 Macrophages Contributes to Cardiac Remodeling after Myocardial Infarction in Rats: a Critical Role of the Calcium Sensing Receptor/NRLP3 Inflammasome”, Liu W, Zhang X, Zhao M, Zhang X, Chi J, Liu Y, Lin F, Fu Y, Ma D, Yin X., Cell Physiol Biochem. [47]	2015	Rats with myocardial infarction		X	X	
3. Cytokines	PubMed	10.1016/j.bbadis.2014.12.023	“γ-Glutamyl cysteine and γ-glutamyl valine inhibit TNF-α signaling in intestinal epithelial cells and reduce inflammation in a mouse model of colitis via allosteric activation of the calcium-sensing receptor”, Zhang H, Kovacs-Nolan J, Kodera T, Eto Y, Mine Y., Biochim Biophys Acta. [48]	2015	Human cells (epithelial cells)		X	X	
2. Inflammation	PubMed	10.3390/ijms19113458	“Combining Calcium Phosphates with Polysaccharides: A Bone-Inspired Material Modulating Monocyte/Macrophage Early Inflammatory Response”, Rammal H, Bour C, Dubus M, Entz L, Aubert L, Gangloff SC, Audonnet S, Bercu NB, Boulmedais F, Mauprivez C, Kerdjoudj H., Int J Mol Sci. [49]	2018	Mammalian cells (THP-1)		X		
2. Inflammation	PubMed	10.1016/j.bbadis.2018.08.020	“Autophagy mediates calcium-sensing receptor-induced TNFα production in human preadipocytes”, Mattar P, Bravo-Sagua R, Tobar N, Fuentes C, Troncoso R, Breitwieser G, Lavandero S, Cifuentes M., Biochim Biophys Acta Mol Basis Dis. [50]	2018	Mammalian cells from liposarcoma		X		
2. Inflammation	PubMed	10.1002/jcp.26490	“Calcium-sensing receptor activates the NLRP3 inflammasome in LS14 preadipocytes mediated by ERK1/2 signaling”, D’Espessailles A, Mora YA, Fuentes C, Cifuentes M., J Cell Physiol. [51]	2018	Human adipocytes cells		X		
3. Cytokines	PubMed	10.3892/ijmm.2018.3924	“Calcium-sensing receptors in human peripheral T lymphocytes and AMI: Cause and effect”, Zeng J, Pan Y, Cui B, Zhai T, Gao S, Zhao Q, Sun Y., Int J Mol Med. [52]	2018	Human cells (T lymphocytes)		X		X
3. Cytokines	PubMed	10.1155/2018/8945850	“Stromal Cell-Derived Factor-1α Alleviates Calcium-Sensing Receptor Activation-Mediated Ischemia/Reperfusion Injury by Inhibiting Caspase-3/Caspase-9-Induced Cell Apoptosis in Rat Free Flaps”, Song L, Gao LN, Wang J, Thapa S, Li Y, Zhong XB, Zhao HW, Xiang XR, Zhang FG, Ji P., Biomed Res Int. [53]	2018	Rat cells (free flap rats (epigastric model))			X	
2. Inflammation	PubMed	10.3390/nu11123072	“Nutritional and Pharmacological Targeting of the Calcium-Sensing Receptor Influences Chemically Induced Colitis in Mice”, Elajnaf T, Iamartino L, Mesteri I, Müller C, Bassetto M, Manhardt T, Baumgartner-Parzer S, Kallay E, Schepelmann M., Nutrients. [54]	2019	Mice with colitis			X	
3. Cytokines	PubMed	10.1152/ajpheart.00308.2019	“MCP-1 mediates ischemia-reperfusion-induced cardiomyocyte apoptosis via MCPIP1 and CaSR”, Zhang W, Zhu T, Chen L, Luo W, Chao J., Am J Physiol Heart Circ Physiol. [55]	2019	Mice with ischemia /reperfusion syndrome		X		
2. Inflammation	PubMed	10.1038/s41467-020-17749-6	“Calcium-sensing receptor-mediated NLRP3 inflammasome response to calciprotein particles drives inflammation in rheumatoid arthritis”, Jäger E, Murthy S, Schmidt C, Hahn M, Strobel S, Peters A, Stäubert C, Sungur P, Venus T, Geisler M, Radusheva V, Raps S, Rothe K, Scholz R, Jung S, Wagner S, Pierer M, Seifert O, Chang W, Estrela-Lopis I, Raulien N, Krohn K, Sträter N, Hoeppener S, Schöneberg T, Rossol M, Wagner U., Nat Commun. [56]	2020	Mice with autoimmune disease (Experimental arthritis). Monocytes from healthy and RA patients/ KO CaSR mice. THP-1/LPS cells		X	X	X
2. Inflammation	PubMed	10.1002/ptr.6777	“Phenol glycosides extract of Fructus Ligustri Lucidi attenuated depressive-like behaviors by suppressing neuroinflammation in hypothalamus of mice”, Feng R, He MC, Li Q, Liang XQ, Tang DZ, Zhang JL, Liu SF, Lin FH, Zhang Y., Phytother Res. [57]	2020	Mice with neuroinflammation		X		
2. Inflammation	PubMed	10.1016/j.mce.2019.110654	“Calcium sensing receptor activation in THP-1 macrophages triggers NLRP3 inflammasome and human preadipose cell inflammation”, D’Espessailles A, Santillana N, Sanhueza S, Fuentes C, Cifuentes M., Mol Cell Endocrinol. [54]	2020	Mammalian cells (THP-1)		X		
3. Cytokines	PubMed	10.1021/acs.jafc.0c04526	“Dietary γ-Glutamyl Valine Ameliorates TNF-α-Induced Vascular Inflammation via Endothelial Calcium-Sensing Receptors”, Guha S, Paul C, Alvarez S, Mine Y, Majumder K., J Agric Food Chem. [58]	2020	Human cells(epithelial cells)		X		
3. Cytokines	PubMed	10.1111/iej.13386	“Mineral trioxide aggregate suppresses pro-inflammatory cytokine expression via the calcineurin/nuclear factor of activated T cells/early growth response 2 pathway in lipopolysaccharide-stimulated macrophages”, Kuramoto M, Kawashima N, Tazawa K, Nara K, Fujii M, Noda S, Hashimoto K, Nozaki K, Okiji T., Int Endod J. [59]	2020	Human cells (RAW264.7 macrophage)		X		
3. Cytokines	PubMed	10.1016/j.cjca.2019.09.026	“Calcium-Sensing Receptor on Neutrophil Promotes Myocardial Apoptosis and Fibrosis After Acute Myocardial Infarction via NLRP3 Inflammasome Activation”, Ren Z, Yang K, Zhao M, Liu W, Zhang X, Chi J, Shi Z, Zhang X, Fu Y, Liu Y, Yin X., Can J Cardiol. [60]	2020	Human cells (PBMC)			X	X
3. Cytokines	PubMed	10.1038/s41419-022-04507-3	“Danger signal extracellular calcium initiates differentiation of monocytes into SPP1/osteopontin-producing macrophages”, Murthy S, Karkossa I, Schmidt C, Hoffmann A, Hagemann T, Rothe K, Seifert O, Anderegg U, von Bergen M, Schubert K, Rossol M., Cell Death Dis. [61]	2022	Human cells (monocytes)		X		X
2. Inflammation	PubMed	10.3389/fimmu.2023.1114129	“Phenylalanine promotes alveolar macrophage pyroptosis via the activation of CaSR in ARDS”, Tang Y, Yu Y, Li R, Tao Z, Zhang L, Wang X, Qi X, Li Y, Meng T, Qu H, Zhou M, Xu J, Liu J., Front Immunol. [62]	2023	In vitro study with mammalian cells		X	X	X
2. Inflammation	PubMed	10.1152/ajpgi.00066.2023	“Alterations in gene expression and microbiome composition upon calcium-sensing receptor deletion in the mouse esophagus”, Abdulnour-Nakhoul SM, Kolls JK, Flemington EK, Ungerleider NA, Nakhoul HN, Song K, Nakhoul NL., Am J Physiol Gastrointest Liver Physiol. [63]	2024	Mouse model of esophagus			X	

**Table 2:** References of reviews included

Keywords used for search strategy	Database	DOI	Reference (Title, Authors, Journal)	Year of publication
3. Cytokines	PubMed	10.1016/s0049-3848(03)00381-5	“TNFalpha regulates renal COX-2 in the rat thick ascending limb (TAL)”, Ferreri NR, McGiff JC, Vio CP, Carroll MA., Thromb Res. [64]	2003
3. Cytokines	PubMed	10.3945/an.111.000745	“Burns: where has all the calcium (and vitamin D) gone?“, Klein GL., Adv Nutr. [65]	2011
3. Cytokines	PubMed	10.1016/j.prostaglandins.2011.11.002	“Eicosanoids and tumor necrosis factor-alpha in the kidney”, Ferreri NR, Hao S, Pedraza PL, Escalante B, Vio CP., Prostaglandins Other Lipid Mediat. [66]	2012
1. Sepsis and septic shock	PubMed	10.1097/SHK.0000000000000261	“Dysregulation of intracellular calcium transporters in animal models of sepsis-induced cardiomyopathy”, Hobai IA, Edgecomb J, LaBarge K, Colucci WS., Shock. [67]	2015
1. Sepsis and septic shock	PubMed	10.3389/fphys.2016.00394	“Calcium-Sensing Receptor Gene: Regulation of Expression”, Hendy GN, Canaff L., Front Physiol. [82]	2016
1. Sepsis and septic shock	PubMed	10.1016/j.semcdb.2015.11.006	“Calcium-sensing receptor, proinflammatory cytokines and calcium homeostasis”, Hendy GN, Canaff L., Semin Cell Dev Biol. [68]	2016
2. Inflammation	PubMed	10.1016/j.pupt.2016.09.010	“Inundation of asthma target research: Untangling asthma riddles”, Singh J, Shah R, Singh D., Pulm Pharmacol Ther. [69]	2016
2. Inflammation	PubMed	10.1016/j.pharmthera.2016.04.002	“Bronchoprotection and bronchorelaxation in asthma: New targets, and new ways to target the old ones”, Pera T, Penn RB., Pharmacol Ther. [70]	2016
2. Inflammation	PubMed	10.1016/j.semcdb.2015.08.006	“The calcium-sensing receptor as a mediator of inflammation”, Klein GL, Castro SM, Garofalo RP., Semin Cell Dev Biol. [71]	2016
2. Inflammation	PubMed	10.3390/biom8030069	“The Role of Calcium in Inflammation-Associated Bone Resorption”, Klein GL., Biomolecules. [72]	2018
3. Cytokines	Google Scholar	10.1016/j.lfs.2018.08.016	“Important roles of the Ca2+-sensing receptor in vascular health and disease”, Guo Y, Yang X, He J, Liu J, Yang S, Dong H., Life sciences. [73]	2018
2. Inflammation	PubMed	10.1080/17476348.2020.1756779	“Calcilytics: a non-steroidal replacement for inhaled steroid and SABA/LABA therapy of human asthma?“, Corrigan CJ., Expert Rev Respir Med. [74]	2020
1. Sepsis and septic shock	PubMed	10.1002/ddr.21797	“Calcium sensing receptor as a novel target for treatment of sepsis induced cardio-renal syndrome: Need for exploring mechanisms”, Yadav S, Gupta K, Deshmukh K, Bhardwaj L, Dahiya A, Krishan P, Singh G., Drug Dev Res. [75]	2021
2. Inflammation	PubMed	10.3390/ijms22052478	“Calcium-Sensing Receptor (CaSR), Its Impact on Inflammation and the Consequences on Cardiovascular Health”, Sundararaman SS, van der Vorst EPC., Int J Mol Sci. [76]	2021
1. Sepsis and septic shock	PubMed	10.3389/fphys.2022.1059369	“The calcium-sensing receptor in inflammation: Recent updates”, Iamartino L, Brandi ML., Front Physiol. [77]	2022
1. Sepsis and septic shock	PubMed	10.1002/ddr.21959	“Pathological role of the calcium-sensing receptor in sepsis-induced hypotensive shock: Therapeutic possibilities and unanswered questions”, Sood A, Singh G, Singh TG, Gupta K., Drug Dev Res. [78]	2022
2. Inflammation	PubMed	10.1021/acs.jafc.2c01712	“Comprehensive Review of γ-Glutamyl Peptides (γ-GPs) and Their Effect on Inflammation Concerning Cardiovascular Health”, Guha S, Majumder K., J Agric Food Chem. [79]	2022

**Fig. 3:**
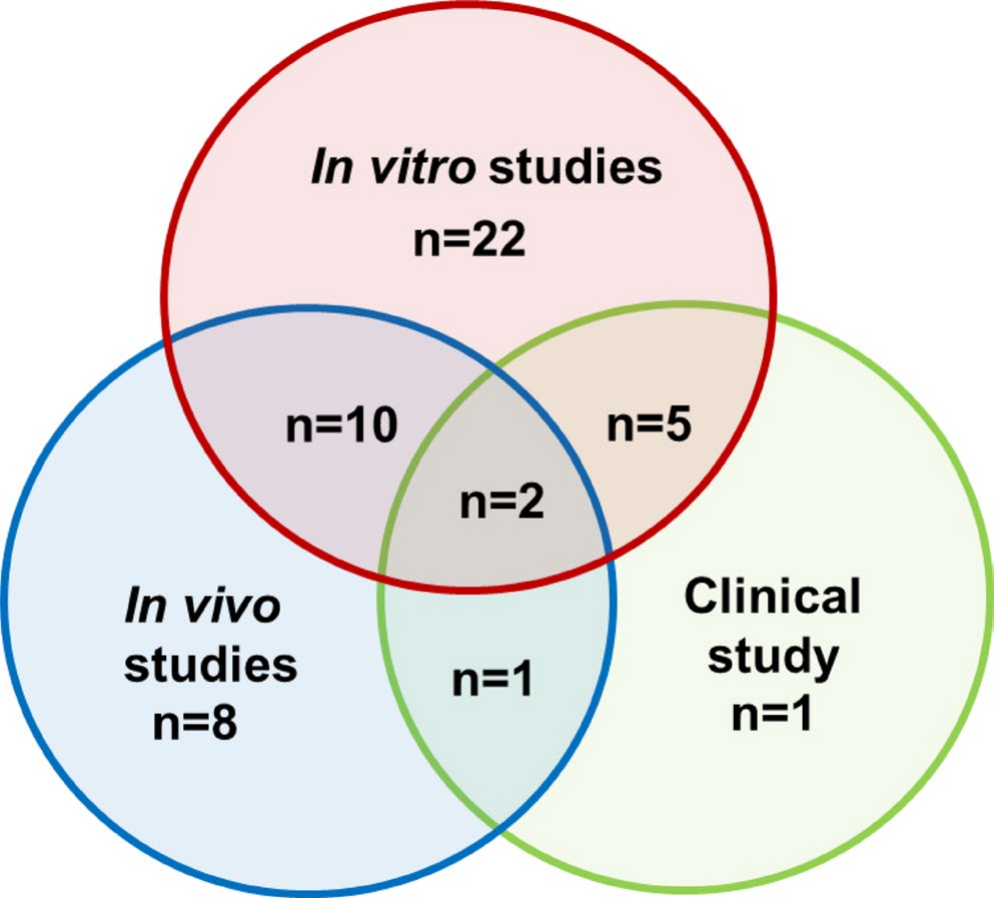
Distribution of the original research articles included based on the type of model studied. Studies (*n* = 49) were categorized by the type of study (in vitro [red], in vivo [blue], clinical [green]). Some of the articles included were based on studies with multiple models (crossed circles). Literature reviews have not been included in this figure

No meta-analyses and no RCTs were identified among the articles retrieved. Most of the 66 articles described in vitro studies and tests with animal models (Fig. 3). Our exclusion criteria would have eliminated case reports, but none were identified at the selection stage. Only 15% of the studies included reported patient-based observational data. Among the articles included, 14 (21%) were within the immediate scope of this review, of which 9 (14%) were identified thanks to keyword-based queries including “septic shock” as a search term.

## In vitro studies addressing septic shock

We identified 9 original in vitro studies investigating the involvement of the CaSR in the pathophysiology of septic shock [18, 20–22, 24–26, 28, 62]. Two of these articles [21, 22] were retrieved using keyword set 1, and are thus considered directly relevant to the core topic of this review **(**Table 1**)**.

Some authors conclude that the CaSR could play a regulatory role in human T lymphocytes under septic conditions. Pharmacological activation of the T lymphocytes CaSR with NPS R-568 recapitulates a sepsis-associated phenotype by up-regulating TRPC3 and TRPC6, thereby driving intracellular calcium influx and apoptosis via the PLC–IP₃ pathway [21]. This upregulation promotes intracellular calcium influx leading to apoptosis via the PLC–IP₃ signaling pathway. A subsequent study indicates that CaSR stimulation of peripheral T lymphocytes also induces secretion of both TNF-α and IL-4, through activation of the Nuclear Factor Kappa B (NF-κB) and Mitogen-Activated Protein Kinase (MAPK) pathways [22].These apparently conclusive results have some limitations: (i) Both studies were published by the same research team in the same journal, raising possible reproducibility concerns. (ii) The specificity of CaSR expression in human T lymphocytes remains insufficiently addressed. This question of specificity of expression is important to interpret the results. In a T lymphocyte cell line (Jurkat JCaM1.6) exposed to staphylococcal superantigens, co-exposure to NPS-2143 – a selective allosteric antagonist of class C G-protein-coupled receptors (GPCRs), including CaSR – reduces IL-2 production by 50% and inhibits CD69 upregulation [25]. Because CaSR is not detected in this T cell line, these findings suggest that the anti-inflammatory effects attributed to NPS-2143 may instead be mediated by an alternative class C GPCR – such as GPRC6A.

In contrast, in innate immune cells, the role of CaSR appears more prominent. In line with Yamaguchi et al. [80] human CD14⁺ monocytes isolated from peripheral blood mononuclear cells (PBMCs) respond robustly to extracellular calcium stimulation by secreting the proinflammatory cytokines IL-1β, IL-1α, IL-6, and TNF-α. This response involves both CaSR and GPRC6A, as demonstrated by siRNA-mediated knockdown experiments [18]. The calcium-dependent proinflammatory response is markedly amplified in the presence of LPS This effect can be attenuated by the CaSR antagonists Calhex 231 and NPS-2143, which both reduce cytokine production by approximately 50% [18]. This in vitro cross-talk between CaSR signaling and LPS-driven inflammation provides a mechanistic basis supporting the potential adjunctive use of calcilytics in treatment of septic shock.

In macrophages, the impact of CaSR on inflammation appears to be phenotype-dependent. Macropinocytosis contributes to macrophage sensing of extracellular danger signals and immune reprogramming, both central features of sepsis. In anti-inflammatory macrophages (CD163⁺, CD200R⁺) derived from Macrophage Colony Stimulating Factor (M-CSF)/IL-4–treated PBMCs, Redka et al. [26] showed that CaSR inhibition by NPS-2143 reduced constitutive macropinocytosis. This effect likely involved interference with Akt membrane translocation. In contrast, proinflammatory macrophages (CD40⁺, CD80⁺) generated with Granulocyte Macrophage - Colony Stimulating Factor (GM-CSF) / Interferon gamma (IFN-γ) / LPS did not display constitutive macropinocytosis, but mounted a transient macropinocytic response following further LPS stimulation ; the effects of calcilytics on this inducible response were not assessed [26]. In line with a proinflammatory role for CaSR in innate immunity, phenylalanine (an allosteric agonist of CaSR that is elevated during acute respiratory distress syndrome) induced NLRP3 inflammasome activation, caspase-1 cleavage, and pyroptosis in murine bone marrow-derived and alveolar macrophages. These effects were reversed by the CaSR antagonist Calhex 231, confirming that the inflammatory pathway is CaSR-dependent [62]). Non-immune barrier cells also contribute to CaSR-mediated inflammation in acute respiratory distress syndrome (ARDS), as demonstrated in an epithelial-endothelial alveolo-capillary model, where exposure to LPS induced CaSR-dependent TNF-α and IL-8 release [62].

Taken together, these observations suggest that CaSR acts as a proinflammatory amplifier across multiple immune cell types, including monocytes and macrophages, and other cell types, but that its modulation yields variable outcomes depending on cell-specific expression patterns, activation states, and co-expression of related GPCRs.

### In vivo studies addressing septic shock

We identified 8 original in vivo research articles directly relevant to CaSR in septic shock [18, 21–25, 27, 62].Three of these articles [21–23] were retrieved using keyword set 1 and are thus considered relevant to the core topic of this review (Table 1).

In two studies, a cecal ligation and puncture (CLP) model of septic shock was studied and found to be linked to overexpression of CaSR in lymphocyte-rich PBMCs. These cells also increased expression of TRPC3 and TRPC6, and apoptosis levels were higher [21, 22]. A complementary in vivo study with a murine model of acute local inflammation examined the impact of CaSR activation. Co-injection of orthosteric CaSR agonists (Ca²⁺, Al³⁺, or Gd³⁺) increased carrageenan-induced footpad swelling (+ 0.2 mm). The effect was diminished in IL-1R⁻/⁻ and Caspase-1⁻/⁻ mice, and completely abolished in GPRC6A⁻/⁻ mice [18]. Further evidence of the involvement of CaSR in inflammation was obtained using a uropathogenic *Escherichia coli*-induced rat orchitis model [27]. The authors report CaSR expression to be upregulated in testicular macrophages compared to the control group (*n* = 5 per group). NLRP3 inflammasome components were similarly overexpressed in these macrophages, contributing to increased interstitial IL-1β secretion. Intratesticular administration of the CaSR inhibitor NPS-2143 (10 mg/kg, one day after infection) reduced local IL-1β concentrations and partially restored testicular tissue integrity (*n* = 5/group).

In C57BL/6 mice with LPS-induced ARDS, pretreatment with the CaSR antagonist NPS-2143 (5 mg/kg) had an anti-inflammatory effect comparable to that of dexamethasone (1 mg/kg). Thus, pretreatment with NPS-2143 significantly reduced pulmonary infiltration of inflammatory cells, MCP-1 expression, neutrophil elastase, and protein levels in bronchoalveolar lavage fluid (BALF) (*n* = 4/group). It also decreased IL-6 and TNF-α levels more than two-fold in both BALF and serum [24]. In a similar ARDS model, but using a larger number of animals (*n* = 35–36), intraperitoneal phenylalanine (a CaSR allosteric agonist) administered before and after the LPS challenge increased BALF IL-1β and IL-18 levels, and exacerbated lung inflammation and macrophage pyroptosis [62]. These effects were associated with a trend for higher mortality. Delayed administration of the CaSR inhibitor Calhex 231 (4 h after the final phenylalanine dose) reduced cytokine levels in BALF, along with the proportion of pyroptotic alveolar macrophages. However, no mortality data were reported for this intervention.

In a toxic shock model using transgenic mice expressing the human antigen-presenting HLA-DR4 molecule (*n* = 20) exposed to staphylococcal enterotoxin B (SEB), treatment with the CaSR inhibitor NPS-2143 significantly prolonged survival (16 h vs. 7.75 h). Enhanced survival was associated with reduced IFN-γ and TNF-α levels, but no effect on IL-2 expression was observed. In contrast to these proinflammatory effects observed in immune cells, oral peptide–mediated activation of the digestive tract CaSR before intraperitoneal LPS challenge was associated with reduced levels of proinflammatory cytokines (IL-1β, IL-6, and TNF-α) in both plasma and the small intestine of mice [23]. The specificity of the oral peptide for CaSR was not assessed, warranting cautious interpretation of these findings.

Taken together, the results of these eight studies suggest that timely inhibition of CaSR may attenuate the inflammatory consequences of septic shock, with most evidence available in the context of ARDS. The results also raise important questions about potential tissue-specific divergences in CaSR-mediated responses. We now critically need interventional data in other models of systemic infection.

### Clinical studies assessing septic shock

We only identified one clinical study directly addressing CaSR and septic shock. It was an observational study conducted at the “Ruijin Hospital Ethics Committee of Shanghai Jiao Tong University School of Medicine”. The authors studied serum phenylalanine concentrations in non-surviving ARDS patients and compared them to concentrations measured in survivors. Circulating phenylalanine has previously been described as a CaSR peptide agonist [62]. In this study, when serum phenylalanine concentrations exceeded the ROC-derived threshold of 3796 µg/mL, survival dropped from 69.70% to 26.67%. However, there are some discrepancies with the raw data (59 patients, 27 survivors), suggesting that 11 patients were either excluded or that imputed values were used [62].

### Studies assessing inflammation and cytokines

Although not sepsis models, several studies explored links between CaSR and inflammatory processes that infer mechanistic pathways relevant to sepsis.

Among the in vitro studies identified in this category, 24 were retrieved using the keyword “cytokines” [11, 19, 29–42, 46–48, 52, 55, 58, 59, 61] and 5 using the keyword “inflammation” [35, 44, 49, 50, 81] .

Among the in vivo studies identified in this category, 7 original articles were retrieved using the keyword “cytokines” [36, 45–48, 53, 60] and 5 were retrieved using “inflammation” [43, 44, 54, 57, 63]). No clinical studies were identified that referred to inflammation, but 5 observational studies referred to “cytokines” [14, 42, 52, 60, 61] (Table 1).

Taken together, the available data indicate that CaSR stimulation triggers predominantly proinflammatory immunomodulatory effects, affecting multiple organs and immune cell types (**Supplementary data**). For example, in monocytes and macrophages, CaSR activation converges on NLRP3 inflammasome signaling and IL-1β–dominant cytokine release. These effects position CaSR as a key amplifier of innate immune activation in inflammatory conditions such as rheumatoid arthritis and asthma [44, 56]. In the cardiovascular system, CaSR signaling has been implicated in myocardial inflammation, endothelial apoptosis, and dysregulated vasoreactivity, processes that mirror sepsis-associated cardiac and vascular dysfunction [60]. In other organs commonly affected in multiple organ failure (lung, kidney, and brain), the available evidence, from experimental models unrelated to sepsis, consistently suggests that CaSR enhances local inflammatory signaling and tissue injury [43] (Table 3). Despite these preclinical data, no randomized controlled trials or interventional human proof-of-concept studies targeting CaSR have been conducted in systemic inflammatory diseases (including asthma and rheumatoid arthritis). (Supplementary data).

**Table 3:** Summary of major concepts and homogeneity of findings across the studies included in this review

Notion	Number of references assessing the notion/both/opposite	References assessing the notion/assessing both/ assessing the opposite
Inhibition of CaSR decreases mortality in sepsis model	1/0/0	[25]
Inhibition of CaSR reduces inflammation in sepsis model	11/0/0	[20, 24, 44–46, 54–56, 58, 59, 81]
CaSR is anti-inflammatory in the intestine	4/0/1	[23, 28, 43, 46] [54]
CaSR is proinflammatory (outside the intestine)	15/1/2	[20–22, 24–27, 44, 50–53, 60, 62, 81] [45] [58, 59]
A proinflammatory context activates the CaSR and increases its expression (outside the intestine)	6/0/1	[20, 22, 25, 27, 48, 60] [42]
CaSR increases the inflammasome pathway	6/0/0	[27, 47, 51, 56, 60, 81]
CaSR is involved in macropinocytosis	2/0/0	[26, 56]

### Inconsistences

Across experimental models, CaSR does not act as a primary inflammatory trigger but rather as a conditional amplifier of inflammation in contexts such as LPS exposure, ischemia–reperfusion, or Damage-Associated Molecular Patterns -rich environments (including elevated extracellular Ca²⁺, polycations, calciprotein particles, or aromatic amino acids such as phenylalanine) [18, 21, 22, 62]. In contrast, gastrointestinal epithelial CaSR activation has been associated with barrier-protective and anti-inflammatory signaling, while CaSR inhibition shows divergent inflammatory effects across immune-driven colitis models [43, 46, 48, 54]. (Table 3). Finally, interpretation of pharmacological studies is complicated by overlapping ligand sensitivity and receptor co-expression within class C GPCRs (particularly GPRC6A) which may account for discordant effects observed in lymphocyte-based models despite minimal CaSR expression.

### CaSR inhibition: use of calcilytic

The use of calcilytic agents across studies provides a potential therapeutic approach and informs on a pharmacokinetic target that may guide future investigations. Among the compounds tested, NPS 2143 was the most frequently studied with heterogeneous administration routes and doses with a descriptive median in vitro concentration of 1µM (CI95% [1–5µM]) to achieve pharmacological effect (Table 4).

**Table 4:** Summary of calcilytic agents, experimental models, and effective doses reported in the literature

Reference	Type of inhibitor	Model	Type of stimulation	Route of administration	Dose used to elicit the effect
“Phenylalanine promotes alveolar macrophage pyroptosis via the activation of CaSR in ARDS”,Tang Y, Yu Y, Li R, Tao Z, Zhang L, Wang X, Qi X, Li Y, Meng T, Qu H, Zhou M, Xu J, Liu J.“,Front Immunol. 2023 Jun 12 [62]	Calhex 231	In vitro	100 ng/ml LPS in cells	In vitro	10µM
	Calhex 231	In vivo	50 µg/mice intrathecal LPS	Intravenous injection in mice	Not reported in mice
“Calcium-sensing receptor-mediated NLRP3 inflammasome response to calciprotein particles drives inflammation in rheumatoid arthritis”,Jäger E, Murthy S, Schmidt C, Hahn M, Strobel S, Peters A, Stäubert C, Sungur P, Venus T, Geisler M, Radusheva V, Raps S, Rothe K, Scholz R, Jung S, Wagner S, Pierer M, Seifert O, Chang W, Estrela-Lopis I, Raulien N, Krohn K, Sträter N, Hoeppener S, Schöneberg T, Rossol M, Wagner U, Nat Commun. 2020 Aug 25 [56]	NPS 2143	In vitro	LPS 100 g/ml	In vitro	10 µM
“Nutritional and Pharmacological Targeting of the Calcium-Sensing Receptor Influences Chemically Induced Colitis in Mice”,“Elajnaf T, Iamartino L, Mesteri I, Müller C, Bassetto M, Manhardt T, Baumgartner-Parzer S, Kallay E, Schepelmann M.“,“Nutrients. 2019 Dec 16 [54]	NPS 2143	In vivo	DSS 3,5%	Orally in mice	10 mg/kg/mouse
“Calcium sensing receptor activation in THP-1 macrophages triggers NLRP3 inflammasome and human preadipose cell inflammation”,D’Espessailles A, Santillana N, Sanhueza S, Fuentes C, Cifuentes M.,Mol Cell Endocrinol. 2020 Feb 5 [81]	NPS 2143	In vitro	LPS 100 ng/ml	In vitro application	1µM
,“Staphylococcal Superantigens Use LAMA2 as a Coreceptor to Activate T Cells”,Li Z, Zeppa JJ, Hancock MA, McCormick JK, Doherty TM, Hendy GN, Madrenas J., J Immunol. 2018 Feb 15 [25]	NPS 2143	In vitro	Staphylococcal enterotoxin E (not reported dose)	In vitro application	10µM
	NPS 2143	In vivo	Biotinylated staphylococcal enterotoxin B 5 µg/mouse	Intraperitoneal injection	30 mg/kg/mouse
“Differential ability of proinflammatory and anti-inflammatory macrophages to perform macropinocytosis”,Redka DS, Gütschow M, Grinstein S, Canton J.,Mol Biol Cell. 2018 Jan 1 [26]	NPS 2143	In vitro	LPS 500 ng/ml	In vitro	10 µM
“Calcium-sensing receptor antagonists abrogate airway hyperresponsiveness and inflammation in allergic asthma”,Yarova PL, Stewart AL, Sathish V, Britt RD Jr, Thompson MA, P Lowe AP, Freeman M, Aravamudan B, Kita H, Brennan SC, Schepelmann M, Davies T, Yung S, Cholisoh Z, Kidd EJ, Ford WR, Broadley KJ, Rietdorf K, Chang W, Bin Khayat ME, Ward DT, Corrigan CJ, T Ward JP, Kemp PJ, Pabelick CM, Prakash YS, Riccardi D.,Sci Transl Med. 2015 Apr 22 [44]	NPS 2143	In vitro	Polycationic peptide poly-L-arginine 300nM (CaSR activator) or spermine	In vitro application	1 µM
	Calhex 231	In vitro	Polycationic peptide poly-L-arginine 300nM (CaSR activator) or spermine	In vitro application	1 µM
	NPS 89,636	In vitro	Polycationic peptide poly-L-arginine 300nM (CaSR activator) + *Alternaria*,* Aspergillus*,* and Dermatophagoides*	In vitro application	100 nM
	NPS 89,636	In vivo	*Alternaria*,* Aspergillus*,* and Dermatophagoides*	Nebulization	3µM per mouse in aerosol volume
“Prokineticin 2 via Calcium-Sensing Receptor Activated NLRP3 Inflammasome Pathway in the Testicular Macrophages of Uropathogenic Escherichia coli-Induced Orchitis”,Su Y, Zhang Y, Hu Z, He L, Wang W, Xu J, Fan Z, Liu C, Zhang H, Zhao K.,Front Immunol. 2020 Oct 23 [27]	NPS 2143	In vitro	LPS (concentration not reported)	In vitro application	Not reported concentration
	NPS 2143	In vivo	50 µL uropathogenic Escherichia coli	Injection in *vas deferens*	10 mg/kg/mouse
“Dietary γ-Glutamyl Valine Ameliorates TNF-α-Induced Vascular Inflammation via Endothelial Calcium-Sensing Receptors”,Guha S, Paul C, Alvarez S, Mine Y, Majumder K.,J Agric Food Chem. 2020 Aug 26 [58]	NPS 2143	In vitro	TNF-α 5ng/mL	In vitro application	1 µM
“Mineral trioxide aggregate suppresses pro-inflammatory cytokine expression via the calcineurin/nuclear factor of activated T cells/early growth response 2 pathway in lipopolysaccharide-stimulated macrophages”,Kuramoto M, Kawashima N, Tazawa K, Nara K, Fujii M, Noda S, Hashimoto K, Nozaki K, Okiji T.,Int Endod J. 2020 Dec [59]	NPS 2143	In vitro	LPS 100ng/mL	In vitro application	5 µM
“Calcium-Sensing Receptor on Neutrophil Promotes Myocardial Apoptosis and Fibrosis After Acute Myocardial Infarction via NLRP3 Inflammasome Activation”,Ren Z, Yang K, Zhao M, Liu W, Zhang X, Chi J, Shi Z, Zhang X, Fu Y, Liu Y, Yin X.,Can J Cardiol. 2020 Jun [60]	Calhex 231	In vitro	Calindol (a CaSR agonist)	In vitro application	5µmol/L
“MCP-1 mediates ischemia-reperfusion-induced cardiomyocyte apoptosis via MCPIP1 and CaSR”,Zhang W, Zhu T, Chen L, Luo W, Chao J.,“Am J Physiol Heart Circ Physiol. 2020 Jan 1 [55]	NPS 2143	In vitro	Cells from ischemia/reperfusion model in mice	In vitro application	Not reported dose
“NPS 2143, a selective calcium-sensing receptor antagonist inhibits lipopolysaccharide-induced pulmonary inflammation”,Lee JW, Park HA, Kwon OK, Park JW, Lee G, Lee HJ, Lee SJ, Oh SR, Ahn KS, Mol Immunol. 2017 Oct [24]	NPS 2143	In vitro	LPS 10 µg/mL	In vitro application	0.5µM
	NPS 2143	In vivo	Intranasal LPS (10 µg dissolved in 50 µl/per mouse)	Oral administration in mice	2.5 mg/kg/mouse
“GPRC6A mediates Alum-induced Nlrp3 inflammasome activation but limits Th2 type antibody responses”,Quandt D, Rothe K, Baerwald C, Rossol M, Sci Rep. 2015 Nov 25 [45]	Calhex 231	In vitro	LPS 100ng/mL and aluminium 40 µg/mL	In vitro application	50 µM
	Calhex 231	In vivo	Intraperitoneal aluminium (1 mg) or ovaprotein (1 mg)	Not reported (intravenous? )	50 µM
“Anti-inflammatory Effects of Poly-L-lysine in Intestinal Mucosal System Mediated by Calcium-Sensing Receptor Activation”,Mine Y, Zhang H., J Agric Food Chem. 2015 Dec 9 [46]	NPS 2143	In vitro	TNF-α 2ng/mL	In vitro application	1 µM
	NPS 2143	In vivo	TNF-α (in vitro concentration not reported) + DSS 5%	Intravenous injection	1 mg/kg
“Extracellular Ca2 + is a danger signal activating the NLRP3 inflammasome through G protein-coupled calcium sensing receptors”,Rossol M, Pierer M, Raulien N, Quandt D, Meusch U, Rothe K, Schubert K, Schöneberg T, Schaefer M, Krügel U, Smajilovic S, Bräuner-Osborne H, Baerwald C, Wagner U.,Nat Commun. 2012 [18]	NPS 2143	In vitro	LPS 100 ng/mL	In vitro application	5µM
	Calhex 231	In vivo	LPS 100 ng/mL + Al-lactate (0.3 mg/per mouse)	Sub plantar co-injection	100µM

For homogeneous datasets, the descriptive median (95% CI) dose was calculated for each calcilytics compound: NPS 2143 in vitro : 1µM (95% CI [1–5µM], bootstrap method; *n* = 8) - Calhex 231 in vitro : 7.5 µM (95% CI [1–50 µM], bootstrap method; *n* = 4) - NPS 89,636 : insufficient data (*n* = 1). Abbreviation: *DSS: dextran sulfate sodium salt – LPS: Lipopolysaccharide*

### Literature reviews

From our searches, 17 review articles were identified (Table 2). Six were directly within the scope of our systematic review and all consistently highlighted the central role played by CaSR in the amplification of inflammatory responses through modulation of cytokine release via NF-κB, ERK1/2, and NLRP3-dependent pathways [67, 68, 75, 77, 78, 82]). The other 11 reviews broadened this pathophysiological framework by suggesting that CaSR signaling might intersect with key mechanisms contributing to septic shock, such as endothelial cell dysfunction, nitric oxide dysregulation, coagulation anomalies, oxidative stress, and microvascular vasoplegia [64–66, 69–74, 76, 79].

Based on this evidence, in septic shock, CaSR appears to be positioned at the crossroads between immune and vascular dysregulation. Nevertheless, as in many of the cases reviewed above, clinical evidence remains scarce.

## Discussion

This first systematic review of the literature relating to the involvement of CaSR in sepsis and septic shock reveals that the field is no very advanced and remains exploratory. This state of advancement is particularly underlined by the absence of RCT. However, there is growing recognition that CaSR has broad immunomodulatory functions. Most of the studies reviewed here relied on in vitro experiments [18, 21, 22, 24–26, 28, 62] alongside some in vivo models [18, 21–25, 27, 62]. Translational and clinical research is now needed to clarify the role of CaSR in sepsis and septic shock and to assess its potential as a therapeutic target. Most of these models rely on LPS challenge. Nevertheless, LPS is thought to be a poor model for clinical sepsis. Large randomized trials of anti-LPS therapies in sepsis showed selective benefits without improvements to mortality [83, 84]. In our review, a single clinical study directly links CaSR to septic shock outcomes through LPS stimuli [62]. However, the robustness of the findings reported is limited due to inconsistencies observed in the reported raw data. Consequently, the clinical rationale for targeting CaSR in this context is weak.

In immune cells, CaSR expression is well established in monocytes and macrophages, where its activation amplifies proinflammatory signaling and supports macropinocytosis [26]). In neutrophils and lymphocytes, evidence of functional CaSR expression is less consistent, and appears to be context-dependent [19, 60]. Lymphocyte-enriched PBMCs harvested following cecal ligation-induced septic shock express CaSR [21, 22], whereas Yamaguchi et al. found that only 4% of unstimulated peripheral blood lymphocytes express this receptor [80]. Given that ~ 80% of CD14⁺ monocytes express CaSR [80], residual CaSR⁺ monocytes may persist despite high-purity T-cell enrichment (e.g., RosetteSep, ~ 96% purity) and confound functional assays. Further evidence of context-dependent expression is provided by Ren et al. [60], who observed that CaSR is expressed by ~ 10% of circulating CD66⁺ neutrophils in healthy individuals, but by almost 99.9% of these cells in patients with acute myocardial infarction.

Emerging evidence indicates that CaSR activation amplifies inflammatory cytokine responses, a hallmark of immune dysregulation in septic shock. In immune cells [20, 25] and in cardiomyocytes [85], activation of CaSR by pathogen-associated molecular patterns – including those found in LPS and staphylococcal toxins – initiates inflammatory signaling cascades that converge on NLRP3 inflammasome assembly, ASC speck formation, and activation of caspase-1, -3, and − 9 [22, 56]. This signaling promotes monocyte recruitment and robust secretion of proinflammatory cytokines such as IL-1β, TNF-α, and IL-6, which contribute to hypercytokinemia, associated with adverse outcomes in septic shock [18, 56].

As IL-1β increases CaSR expression in several cells, its upregulation may aggravate the proinflammatory state by engaging the NLRP3 pathway in a self-amplifying loop [29, 31, 82]. Whether this loop is constitutively operational in immune cells remains to be established; however, CaSR upregulation in PBMCs after cecal ligation and puncture (CLP) supports its existence in septic shock [21, 22]. The possibility that CaSR activation or its overexpression may be associated with increased mortality in patients with septic shock represents an important area of investigation. In this context, our group is currently conducting an observational clinical study of the kinetics of CaSR expression modulation in monocytes from patients with septic shock (ClinicalTrials.gov identifier: NCT06853340).

Given the limited therapeutic options in septic shock, CaSR inhibition appears to be a promising complementary approach that could either be used alongside current immunomodulatory strategies or as an alternative in patients not responding to standard care. Preclinical studies using calcilytics such as NPS-2143 indicate that CaSR inhibition can reduce NLRP3 inflammasome activation, diminish proinflammatory cytokine production, and interrupt the self-perpetuating inflammatory loop, thus limiting immune cell infiltration and preserving tissue integrity [18, 24, 25, 27]. These effects have been observed in vivo in models of toxin-induced ARDS [24, 62], and in *Staphylococcus aureus*-associated toxic shock syndrome [25]. In both cases, a trend for potential mortality reduction was observed. Nevertheless, we lack clinically relevant in vivo models of septic shock incorporating a live bacterial challenge that could be used to validate the therapeutic potential of calcilytics.

The pathophysiology of septic shock is also characterized by profound endothelial dysfunction and impaired vasoreactivity. The role of CaSR in septic shock-associated vasoplegia has not yet been examined, but findings from relevant in vitro systems hint at a possible mechanistic link. For example, in models of circulatory shock, CaSR dysregulation appears to modulate vascular tone and amplify ischemia-reperfusion injury through nitric oxide (NO)-mediated mechanisms. Data from non-septic models indicate that CaSR activation promotes endothelium-dependent vasodilation through TRPV4-TRPC1–mediated calcium influx and subsequent eNOS activation, particularly in mesenteric vascular beds [86, 87]. Other studies implicate CaSR signaling in in vitro myocardial ischemia-reperfusion injury, and the associated oxidative stress, mitochondrial dysfunction, and endothelial damage [53, 55]. Although limited data are available regarding the hemodynamic effects of CaSR inhibition, a recent study in normotensive rats reported modest elevation of mean arterial pressure following NPS-2143 monotherapy [78, 88]. Although the overall level of evidence remains low, CaSR blockade appears to confer several mechanistic advantages in sepsis-associated conditions. Its anti-inflammatory, anti-vasoplegic, and cardioprotective effects, and as reviewed above, alleviation of respiratory distress, acute kidney injury, and critical illness polyneuropathy mark it out as an interesting therapeutic approach in sepsis.

Several calcilytics have already been clinically evaluated for other conditions: NPSP-795 (intravenous) was administered to five adults with autosomal dominant hypocalcemia type 1, where it was generally safe and well tolerated, resulting in dose-dependent increases in plasma PTH without destabilizing ionized calcium levels [89]. Encaleret (oral) was tested in 13 ADH1 patients in a phase 2b study. It normalized serum calcium and PTH while reducing hypercalciuria and preserving renal function, with no serious adverse events [90]. Ronacaleret was used to treat low bone mineral density in 34 postmenopausal women. Dose‑dependent increases in serum PTH and calcium, as well as urinary calcium excretion were recorded, and the treatment was generally well tolerated, with mainly mild gastrointestinal and neurological symptoms reported [91]. Although calcilytics appear safe outside sepsis, their use in septic patients warrants caution, particularly because of the risks of hypercalcemia and cardiac arrhythmias [92]. The debated proinflammatory role of CaSR in the gastrointestinal tract should also be considered in patients with peritonitis [43, 46, 54].

Potential interactions between CaSR-targeted therapies and existing interventions in septic shock should be considered carefully. As CaSR activation is modulated by extracellular calcium and magnesium, and calcium and magnesium are commonly administered in critical care, variations in supplementation may influence CaSR activity and downstream inflammatory signaling [93, 94]. Similarly, certain amino acids and dietary peptides act as positive allosteric modulators of CaSR, potentially modifying its responsiveness to pharmacological inhibition [95, 96]. Notably, dipeptides are known to accumulate in acute lung injury and may be influenced by high-protein feeding regimens [62]. This effect could shed light on the mechanisms underlying the lack of benefit observed during randomized trials using hyperprotein strategies as treatment for septic shock [97]. Similarly, aminoglycosides (antibiotics with a debated role in sepsis) [98] are known to interact with the extracellular domain of CaSR. This binding could potential interfere with CaSR modulation strategies [99], and might partly account for the lack of clear benefit of routine administration of aminoglycosides during treatment of septic shock [100]. Finally, little is known about potential pharmacodynamic interactions with concurrent immunomodulatory agents, such as corticosteroids or IL-6 inhibitors, but the net inflammatory response could be modified.

If septic shock also disrupts CaSR activity at the cellular level, this could mechanistically link immune dysregulation to the frequent observation of perturbed calcium-phosphate metabolism. Excessive activation of the parathyroid CaSR could cause relative hypoparathyroidism [101], with frequent hypocalcemia and impaired hydroxylation of 25(OH) vitamin D₃ (producing 1,25(OH)₂ vitamin D₃) [8]. Currently, calcium-phosphate abnormalities in septic shock are only managed through replacement strategies when deficits are measured. This approach may be counterproductive, as the effects of calcium supplementation in sepsis remain controversial: high-dose calcium worsens outcomes in murine models [102], whereas some observational clinical data suggest survival benefits in hypocalcemic patients [103]. Similarly, although vitamin D supplementation should be beneficial in theory, the VITdAL -ICU trial RCT showed no significant difference in survival among critically ill patients receiving vitamin D3 supplementation [104]. Furthermore, in a murine model of endotoxemia, pretreatment with paricalcitol (an active vitamin D agonist) reduced myocardial inflammation, NF-κB activation, endothelial adhesion molecule expression, and vascular leakage, suggesting endothelial protection through immuno-inflammatory modulation [105]. Collectively, these observations raise the prospect of a paradigm shift from simple supplementation to modulation of calcium-phosphate metabolism, with early use of active therapeutics.

The main limitation of this review is the lack of randomized controlled trials, resulting in a qualitative synthesis and limiting formal evaluation of inter-study heterogeneity. Consequently, conclusions are restricted to preclinical evidence. As the literature search was completed at the end of 2024, the studies reviewed did not include anything published thereafter. An updated search performed in September 2025 using the same keyword sets identified four additional original research articles [106–109]. Most of these studies confirm existing concepts, but one provides novel mechanistic insights, demonstrating that CaSR-mediated NLRP3 activation in human umbilical vein endothelial cells relies on β1-integrin signaling [106].

This review has several strengths: (i) development of a systematic design based on a comprehensive multi-database literature search with predefined eligibility criteria; (ii) use of independent screening and data extraction by multiple reviewers, minimizing bias and enhancing objectivity; and (iii) application of a statistical approach that strengthens the robustness of the narrative synthesis.

For clinicians, this systematic review offers a critical appraisal of current evidence linking CaSR signaling to sepsis. Preclinical data indicate that sepsis is associated with CaSR upregulation, extending its role beyond calcium–phosphate homeostasis toward amplification of proinflammatory responses. Experimental inhibition of CaSR in sepsis has yielded encouraging signals, particularly in models of acute respiratory distress syndrome and toxin-induced shock. Positioning CaSR as both a pathophysiological marker and a potential therapeutic target may help link experimental findings to bedside challenges, and should be instrumental in the design of future interventional studies involving critically ill patients.

## Supplementary Information

Below is the link to the electronic supplementary material.

Supplementary Material 1

## Data Availability

The data that support the findings of this systematic review are available upon reasonable request by contacting the corresponding author.
