# Supplementary material for: The calcium-sensing receptor in sepsis and septic shock, mechanistic pathways and translational perspectives: a systematic review
Source: Inflamm Res. 2026 Apr 17;75(1):99. doi: 10.1007/s00011-026-02227-4 (PMC13090257; doi:10.1007/s00011-026-02227-4)
Supplement: Supplementary file 1 — Supplementary Material 1 [file 11_2026_2227_MOESM1_ESM.docx]

**Supplementary material**

*CaSR as proinflammatory in immune cells*

As detailed in the review, CaSR is reported to be functionally expressed in multiple monocyte-derived cell types, where it acts as a central regulator of inflammatory responses. In THP-1 cells, its protein and functional expression appears to depend on the degree of differentiation toward a macrophage-like phenotype [1]. In undifferentiated THP-1 cells, CaSR activation appears to inconsistently trigger IL-1β secretion [2]. For clinical samples, CaSR expression by monocytes can be assessed longitudinally by flow cytometry [3]. In PBMCs, CaSR mediates the internalization of calciprotein particles (CPPs) via macropinocytosis. This process is dependent on extracellular calcium, phosphate, and fetuin-A. CaSR activation on monocytes leads to lysosomal stress and NLRP3 inflammasome activation – induced by CaSR via both PLC–IP₃ signaling and cAMP downregulation [4] along with IL-1β release [5] as well as secretion of TNF-α, IL-6, IL-8, and MCP-1 at moderate levels, and IL-10 at low levels [6].

The proinflammatory response to CaSR is markedly amplified in monocytes from patients with rheumatoid arthritis, a condition that shares features with cytokine storm [5]. CRISPR-Cas9 studies further demonstrate that extracellular Ca²⁺ signaling through CaSR drives monocyte-to-macrophage differentiation along a non-classical inflammatory trajectory. These "Ca²⁺-macrophages" release high levels of IL-1β in response to LPS/Ca²⁺ co-stimulation and display increased invasiveness, independent of classical M1/M2 polarization [7]. The IL-1β secreted by CaSR-activated monocytes can trigger paracrine inflammatory cascades, notably promoting inflammation in preadipocytes [8].

Finally, although most data support a proinflammatory role for CaSR in monocyte-derived cells, Kuramoto et al. [9] report reduced IL-1α and IL-6 mRNA levels in RAW264.7 cells exposed to mineral trioxide aggregate extracts. These effects can be reversed by the calcilytic NPS-2143. These apparently contradictory findings should be considered with care as the study has some limitations, notably the pre-osteoclast lineage and transcript-only outcomes.

In summary, although CaSR emerges as a key proinflammatory mediator in most models, no therapeutic intervention has yet been reported in humans, not even in the treatment of rheumatoid arthritis.

*Link between CaSR and Myocardial Inflammation*

CaSR has emerged as a key mediator of inflammatory responses in myocardial infarction (MI), which shares converging pathways with sepsis, such as damage-induced innate immune activation and ischemia-reperfusion injury. In a cohort of 40 patients with acute MI, neutrophil expression of CaSR, NLRP3, and caspase-1 was significantly increased. Treatment with Calhex-231 reduced NLRP3 activation and IL-1β release, highlighting its therapeutic potential [10]. Similarly, in T cell-enriched PBMCs (≥90%), CaSR expression was increased by 70% in patients with acute MI [11]. Silencing CaSR in extracted T lymphocytes reduced the secretion of both pro- and anti-inflammatory cytokines, including IL-4, IL-6, IL-10, and TNF-α [11] . Surface CaSR expression on CD14+ PBMCs was also found to be elevated in individuals with ischemic heart disease compared to those without [12].

In rats, MI induces overexpression of the CaSR and the NLRP3 inflammasome across all macrophage subpopulations, both in the myocardium and in peritoneal macrophages. However, *in vitro* activation of CaSR triggers NLRP3-dependent IL-1β secretion only in polarized proinflammatory M1 macrophages. In co-cultures, this IL-1β triggers enhanced cardiac fibroblast remodeling [2]. Converging evidence suggests that CaSR is functionally expressed in cardiomyocytes. Ischemia-reperfusion in cardiac cells (HL-1 cell line and primary cultured neonatal mouse ventricular cardiomyocytes) induces MCP-1–mediated CaSR-dependent apoptosis, which is reduced by CaSR blockade [13]).

We now require *in vivo* and clinical investigations with pharmacological CaSR antagonists to establish their potential cardioprotective effects in a context of MI.

*Link between CaSR and Vascular Dysfunction*

Under ischemia-reperfusion conditions, CaSR is phosphorylated in vascular endothelial cells, triggering increased rates of caspase-dependent apoptosis leading to impaired microvascular integrity. Pharmacological inhibition of CaSR with calcilytic NPS-2143 reduces endothelial apoptosis, enhances neovascularization, and improves overall tissue viability in surgical free flaps [14]. However, *in vitro* findings suggest that NPS-2143 may also blunt the anti-inflammatory effects of γ-glutamyl valine on human aortic endothelial cells, revealing potential context-dependent limitations of CaSR antagonism [15].

Beyond inflammation, CaSR contributes to regulating vascular tone. Its activation in endothelial cells triggers the opening of intermediate-conductance calcium-activated potassium (IKCa) channels, leading to endothelial hyperpolarization that is transmitted to vascular smooth muscle cells, promoting vasodilation [16]. It is possible that during systemic inflammation or shock, this mechanism could contribute to inappropriate vasodilation. This hypothesis will now need to be tested with a specific septic model and clinical intervention.

*CaSR and inflammation in obesity*

Obesity is associated with increased sepsis-related mortality, with inflammation within adipose tissue potentially playing a contributing role. *In vitro* studies identified CaSR as a potential amplifier of adipose inflammation. Thus, in human preadipocytes, CaSR activation induces TNF-α production via autophagy-dependent mechanisms [17] and promotes NLRP3 inflammasome activation in LS14 adipocytes through ERK1/2 signaling [18]. These findings suggest that CaSR may contribute to the proinflammatory priming of adipose tissue, and this obesity-associated inflammation may be the reason for worse outcomes in sepsis in people with obesity.

*CaSR and kidney inflammation*

*In vitro* studies with kidney cells suggest that CaSR may contribute to a proinflammatory amplification loop in this tissue. For example, in tubular epithelial cells, CaSR activation triggers the production of proinflammatory cytokines such as IL-8 and MCP-1 through ERK1/2-dependent signaling [19,20]. In addition, in medullary thick ascending limb (mTAL) cells, stimulation of CaSR inhibits apical chloride reabsorption and increases TNF-α and prostaglandin E2 synthesis through the NFAT pathway [19–21]. CaSR expression in kidney cells is upregulated by IL-1β through NF-κB–dependent promoter activation. This potential positive feedback loop may then sustain renal inflammation [22].

Although mechanistically plausible, we currently lack clinical and *in vivo* evidence of these proinflammatory loops, particularly in the context of sepsis-induced acute kidney injury.

*CaSR and brain inflammation*

Emerging evidence indicates that CaSR may also contribute to central nervous system inflammation, expanding its known immunomodulatory functions. In a murine model of infection-driven neuroinflammation, pharmacological inhibition of CaSR using phenolic glycosides markedly reduces IL-1β production in the hypothalamus [23]. Similarly, in human astrocyte cultures, the calcilytic agent NPS 89636 inhibits cytokine-induced nitric oxide production when applied during a (very) narrow time window (24–24.5 h post-stimulation) [24]. These data provide a broader perspective on CaSR blockade, extending its impact to critical illness-associated neuropathy, another field that deserves further exploration.

*CaSR and pulmonary inflammation*

CaSR expression is not only relevant in acute respiratory infection models. Indeed, its expression is reported to be increased in airway smooth muscle cells in patients with asthma [25] and in experimental models of pulmonary hypertension [26]. Extracellular calcium or polycation-driven CaSR activation leads to bronchial hyperresponsiveness and local inflammation, both of which are effectively suppressed by calcilytics *in vivo* [25] . In a pulmonary hypertension *in vivo* model, pharmacological CaSR inhibition by NPS-2143 mitigates pulmonary vascular remodeling and prevents right ventricular hypertrophy [26]. Taken together, these pulmonary properties provide a favorable rationale for exploring the usefulness of calcilytics in intubated patients with sepsis.

*CaSR and gastrointestinal inflammation*

In digestive models, modulation of CaSR (through changes in expression, activation, or inhibition) is associated with heterogeneous inflammatory responses covering the full pro- to anti- spectrum. In both colon tissue from a murine model of induced colitis and in Caco-2 digestive epithelial cells *in vitro*, activation of CaSR by γ-glutamyl peptides decreases TNF-α, IL-1β, IL-17, and IL-6 levels, and increases IL-10 levels [27]. Similar anti-inflammatory effects were achieved with poly-L-lysine, and could be abolished by the use of calcilytics, confirming the involvement of CaSR. In line with these results, loss of CaSR function in the intestinal epithelium in specific knockout mice led to a marked increase in intestinal inflammation compared to that observed in wild-type controls [28,29].

The uniformity of the anti-inflammatory effect of CaSR within the gastrointestinal tract has been questioned based on results from other *in vivo* studies. Thus, in the DSS colitis mouse model, compared to a vehicle control, the CaSR antagonist NPS-2143 significantly reduces the cumulative inflammation score and infiltration of inflammatory cells [30]. Similarly, conditional deletion of CaSR in the murine esophageal epithelium disrupts barrier integrity, alters the microbiome, and triggers CXCR2-driven inflammation [31].

The divergence of these experimental data raises concerns on the relevance of pharmacological CaSR blockade in sepsis with peritonitis. As a result, patients with this presentation should be regarded as a distinct subgroup and offered adapted care.

***Supplementary material references***

1. Xi Y, Li H, Zhang W, Wang L, Zhang L, Lin Y, et al. The functional expression of calcium-sensing receptor in the differentiated THP-1 cells. Mol Cell Biochem. 2010;342:233–40. https://doi.org/10.1007/s11010-010-0489-3

2. Liu W, Zhang X, Zhao M, Zhang X, Chi J, Liu Y, et al. Activation in M1 but not M2 Macrophages Contributes to Cardiac Remodeling after Myocardial Infarction in Rats: a Critical Role of the Calcium Sensing Receptor/NRLP3 Inflammasome. Cellular Physiology and Biochemistry. 2015;35:2483–500. https://doi.org/10.1159/000374048

3. Paccou J, Boudot C, Mary A, Kamel S, Drüeke TB, Fardellone P, et al. Determination and modulation of total and surface calcium-sensing receptor expression in monocytes in vivo and in vitro. PLoS One. 2013;8:e74800. https://doi.org/10.1371/journal.pone.0074800

4. Lee G-S, Subramanian N, Kim AI, Aksentijevich I, Goldbach-Mansky R, Sacks DB, et al. The calcium-sensing receptor regulates the NLRP3 inflammasome through Ca2+ and cAMP. Nature. 2012;492:123–7. https://doi.org/10.1038/nature11588

5. Jäger E, Murthy S, Schmidt C, Hahn M, Strobel S, Peters A, et al. Calcium-sensing receptor-mediated NLRP3 inflammasome response to calciprotein particles drives inflammation in rheumatoid arthritis. Nat Commun. Nature Publishing Group; 2020;11:4243. https://doi.org/10.1038/s41467-020-17749-6

6. Rammal H, Bour C, Dubus M, Entz L, Aubert L, Gangloff SC, et al. Combining Calcium Phosphates with Polysaccharides: A Bone-Inspired Material Modulating Monocyte/Macrophage Early Inflammatory Response. Int J Mol Sci. 2018;19:3458. https://doi.org/10.3390/ijms19113458

7. Murthy S, Karkossa I, Schmidt C, Hoffmann A, Hagemann T, Rothe K, et al. Danger signal extracellular calcium initiates differentiation of monocytes into SPP1/osteopontin-producing macrophages. Cell Death Dis. Nature Publishing Group; 2022;13:1–15. https://doi.org/10.1038/s41419-022-04507-3

8. D’Espessailles A, Santillana N, Sanhueza S, Fuentes C, Cifuentes M. Calcium sensing receptor activation in THP-1 macrophages triggers NLRP3 inflammasome and human preadipose cell inflammation. Mol Cell Endocrinol. 2020;501:110654. https://doi.org/10.1016/j.mce.2019.110654

9. Kuramoto M, Kawashima N, Tazawa K, Nara K, Fujii M, Noda S, et al. Mineral trioxide aggregate suppresses pro-inflammatory cytokine expression via the calcineurin/nuclear factor of activated T cells/early growth response 2 pathway in lipopolysaccharide-stimulated macrophages. Int Endod J. 2020;53:1653–65. https://doi.org/10.1111/iej.13386

10. Ren Z, Yang K, Zhao M, Liu W, Zhang X, Chi J, et al. Calcium-Sensing Receptor on Neutrophil Promotes Myocardial Apoptosis and Fibrosis After Acute Myocardial Infarction via NLRP3 Inflammasome Activation. Can J Cardiol. 2020;36:893–905. https://doi.org/10.1016/j.cjca.2019.09.026

11. Zeng J, Pan Y, Cui B, Zhai T, Gao S, Zhao Q, et al. Calcium‑sensing receptors in human peripheral T lymphocytes and AMI: Cause and effect. International Journal of Molecular Medicine. Spandidos Publications; 2018;42:3437–46. https://doi.org/10.3892/ijmm.2018.3924

12. Malecki R, Fiodorenko-Dumas Z, Jakobsche-Policht U, Malodobra M, Adamiec R. ALTERED MONOCYTE CALCIUM-SENSING RECEPTOR EXPRESSION IN PATIENTS WITH TYPE 2 DIABETES MELLITUS AND ATHEROSCLEROSIS.

13. Zhang W, Zhu T, Chen L, Luo W, Chao J. MCP-1 mediates ischemia-reperfusion-induced cardiomyocyte apoptosis via MCPIP1 and CaSR. Am J Physiol Heart Circ Physiol. 2020;318:H59–71. https://doi.org/10.1152/ajpheart.00308.2019

14. Song L, Gao L-N, Wang J, Thapa S, Li Y, Zhong X-B, et al. Stromal Cell-Derived Factor-1*α* Alleviates Calcium-Sensing Receptor Activation-Mediated Ischemia/Reperfusion Injury by Inhibiting Caspase-3/Caspase-9-Induced Cell Apoptosis in Rat Free Flaps. BioMed Research International. Hindawi; 2018;2018:e8945850. https://doi.org/10.1155/2018/8945850

15. Guha S, Paul C, Alvarez S, Mine Y, Majumder K. Dietary γ-Glutamyl Valine Ameliorates TNF-α-Induced Vascular Inflammation via Endothelial Calcium-Sensing Receptors. J Agric Food Chem. 2020;68:9139–49. https://doi.org/10.1021/acs.jafc.0c04526

16. Weston AH, Absi M, Ward DT, Ohanian J, Dodd RH, Dauban P, et al. Evidence in favor of a calcium-sensing receptor in arterial endothelial cells: studies with calindol and Calhex 231. Circ Res. 2005;97:391–8. https://doi.org/10.1161/01.RES.0000178787.59594.a0

17. Mattar P, Bravo-Sagua R, Tobar N, Fuentes C, Troncoso R, Breitwieser G, et al. Autophagy mediates calcium-sensing receptor-induced TNFα production in human preadipocytes. Biochimica et Biophysica Acta (BBA) - Molecular Basis of Disease. 2018;1864:3585–94. https://doi.org/10.1016/j.bbadis.2018.08.020

18. D’Espessailles A, Mora YA, Fuentes C, Cifuentes M. Calcium-sensing receptor activates the NLRP3 inflammasome in LS14 preadipocytes mediated by ERK1/2 signaling. Journal of Cellular Physiology. 2018;233:6232–40. https://doi.org/10.1002/jcp.26490

19. Abdullah HI, Pedraza PL, Hao S, Rodland KD, McGiff JC, Ferreri NR. NFAT regulates calcium-sensing receptor-mediated TNF production. American Journal of Physiology-Renal Physiology. American Physiological Society; 2006;290:F1110–7. https://doi.org/10.1152/ajprenal.00223.2005

20. Abdullah HI, Pedraza PL, McGiff JC, Ferreri NR. Calcium-sensing receptor signaling pathways in medullary thick ascending limb cells mediate COX-2-derived PGE2 production: functional significance. American Journal of Physiology-Renal Physiology. American Physiological Society; 2008;295:F1082–9. https://doi.org/10.1152/ajprenal.90316.2008

21. Wang D, Pedraza PL, Abdullah HI, McGiff JC, Ferreri NR. Calcium-sensing receptor-mediated TNF production in medullary thick ascending limb cells. American Journal of Physiology-Renal Physiology. American Physiological Society; 2002;283:F963–70. https://doi.org/10.1152/ajprenal.00108.2002

22. Canaff L, Hendy GN. Calcium-sensing Receptor Gene Transcription Is Up-regulated by the Proinflammatory Cytokine, Interleukin-1β: ROLE OF THE NF-κB PATHWAY AND κB ELEMENTS*. Journal of Biological Chemistry. 2005;280:14177–88. https://doi.org/10.1074/jbc.M408587200

23. Feng R, He M-C, Li Q, Liang X-Q, Tang D-Z, Zhang J-L, et al. Phenol glycosides extract of Fructus Ligustri Lucidi attenuated depressive-like behaviors by suppressing neuroinflammation in hypothalamus of mice. Phytother Res. 2020;34:3273–86. https://doi.org/10.1002/ptr.6777

24. Dal Pra I, Chiarini A, Nemeth EF, Armato U, Whitfield JF. Roles of Ca2+ and the Ca2+-sensing receptor (CASR) in the expression of inducible NOS (nitric oxide synthase)-2 and its BH4 (tetrahydrobiopterin)-dependent activation in cytokine-stimulated adult human astrocytes. Journal of Cellular Biochemistry. 2005;96:428–38. https://doi.org/10.1002/jcb.20511

25. Yarova PL, Stewart AL, Sathish V, Britt RD, Thompson MA, Lowe APP, et al. Calcium-sensing receptor antagonists abrogate airway hyperresponsiveness and inflammation in allergic asthma. Sci Transl Med. 2015;7:284ra60. https://doi.org/10.1126/scitranslmed.aaa0282

26. Yamamura A, Guo Q, Yamamura H, Zimnicka AM, Pohl NM, Smith KA, et al. Enhanced Ca2+-Sensing Receptor Function in Idiopathic Pulmonary Arterial Hypertension. Circulation Research. American Heart Association; 2012;111:469–81. https://doi.org/10.1161/CIRCRESAHA.112.266361

27. Zhang H, Kovacs-Nolan J, Kodera T, Eto Y, Mine Y. γ-Glutamyl cysteine and γ-glutamyl valine inhibit TNF-α signaling in intestinal epithelial cells and reduce inflammation in a mouse model of colitis via allosteric activation of the calcium-sensing receptor. Biochim Biophys Acta. 2015;1852:792–804. https://doi.org/10.1016/j.bbadis.2014.12.023

28. Mine Y, Zhang H. Anti-inflammatory Effects of Poly-L-lysine in Intestinal Mucosal System Mediated by Calcium-Sensing Receptor Activation. J Agric Food Chem. 2015;63:10437–47. https://doi.org/10.1021/acs.jafc.5b03812

29. Cheng SX, Lightfoot YL, Yang T, Zadeh M, Tang L, Sahay B, et al. Epithelial CaSR Deficiency Alters Intestinal Integrity and Promotes Proinflammatory Immune Responses. FEBS Lett. 2014;588:4158–66. https://doi.org/10.1016/j.febslet.2014.05.007

30. Elajnaf T, Iamartino L, Mesteri I, Müller C, Bassetto M, Manhardt T, et al. Nutritional and Pharmacological Targeting of the Calcium-Sensing Receptor Influences Chemically Induced Colitis in Mice. Nutrients. Multidisciplinary Digital Publishing Institute; 2019;11:3072. https://doi.org/10.3390/nu11123072

31. Abdulnour-Nakhoul SM, Kolls JK, Flemington EK, Ungerleider NA, Nakhoul HN, Song K, et al. Alterations in gene expression and microbiome composition upon calcium-sensing receptor deletion in the mouse esophagus. Am J Physiol Gastrointest Liver Physiol. 2024;326:G438–59. https://doi.org/10.1152/ajpgi.00066.2023
